# LPS Down-Regulates Specificity Protein 1 Activity by Activating NF-κB Pathway in Endotoxemic Mice

**DOI:** 10.1371/journal.pone.0130317

**Published:** 2015-06-23

**Authors:** Xiaobing Ye, Hong Liu, Yong-Sheng Gong, Shu Fang Liu

**Affiliations:** 1 Centers for Heart and Lung Research, and Pulmonary and Critical Care Medicine, the Feinstein Institute for Medical Research, Manhasset, New York, United States; 2 Institute of Hypoxia Medicine, Wenzhou Medical University, Wenzhou, China; Universidade de São Paulo, BRAZIL

## Abstract

**Background:**

Specificity protein (Sp) 1 mediates the transcription of a large number of constitutive genes encoding physiological mediators. NF-κB mediates the expression of hundreds of inducible genes encoding pathological mediators. Crosstalk between Sp1 and NF-κB pathways could be pathophysiologically significant, but has not been studied. This study examined the crosstalk between the two pathways and defined the role of NF-κB signaling in LPS-induced down-regulation of Sp1 activity.

**Methods and Main Findings:**

Challenge of wild type mice with *samonelia enteritidis* LPS (10 mg/kg, i.p.) down-regulated Sp1 binding activity in lungs in a time-dependent manner, which was concomitantly associated with an increased NF-κB activity. LPS down-regulates Sp1 activity by inducing an LPS inducible Sp1-degrading enzyme (LISPDE) activity, which selectively degrades Sp1 protein, resulting in Sp1 down-regulation. Blockade of NF-κB activation in mice deficient in NF-κB p50 gene (NF-κB-KO) suppressed LISPDE activity, prevented Sp1 protein degradation, and reversed the down-regulation of Sp1 DNA binding activity and eNOS expression (an indicator of Sp1 transactivation activity). Inhibition of LISPDE activity using a selective LISPDE inhibitor mimicked the effects of NF-κB blockade. Pretreatment of LPS-challenged WT mice with a selective LISPDE inhibitor increased nuclear Sp1 protein content, restored Sp1 DNA binding activity and reversed eNOS protein down-regulation in lungs. Enhancing tissue level of Sp1 activity by inhibiting NF-κB-mediated Sp1 down-regulation increased tissue level of IL-10 and decreased tissue level of TNF- αin the lungs.

**Conclusions:**

NF-κB signaling mediates LPS-induced down-regulation of Sp1 activity. Activation of NF-κB pathway suppresses Sp1 activity and Sp1-mediated anti-inflammatory signals. Conversely, Sp1 signaling counter-regulates NF-κB-mediated inflammatory response. Crosstalk between NF-κB and Sp1 pathways regulates the balance between pro- and anti-inflammatory cytokines.

## Introduction

Specificity protein (Sp) 1 is a ubiquitous transcription factor that belongs to Sp family of proteins consisting of at least 9 members, Sp1-9 [1, 2]. Sp1 mediates the transcription of a large number of constitutive genes, including genes encoding neurotransmitters, and their transporters and receptors [3–5], signaling molecules [6], ion channels [7, 8], physiological enzymes, such as endothelial nitric oxide synthase (eNOS) [9], as well as enzymes participating in fat, sugar and protein metabolism [10–12]. Sp1 pathway may play critical roles in diverse physiological processes and in maintaining normal organ function.

Sp1 activity is essential for the expression of most growth factors and their receptors, including vascular endothelial growth factor and its receptors [13, 14], fibroblast growth factor and its receptors [15, 16], and transforming growth factor receptor [17]. Sp1 mediates gene transcription of extracellular matrix [18, 19] as well as enzymes involving in matrix metabolism. These gene products are important for wound healing and tissue repair, suggesting that the Sp1 pathway may regulates tissue repair and wound healing.

Sp1 mediates transcriptional expression of multiple anti-inflammatory genes, including IL-10, suppressor of cytokine signaling 3 and peroxisome proliferator activated receptor- [20–22], suggesting that Sp1 activity may play an anti-inflammatory role.

Overall, the Sp1 pathway is a major constitutive signaling pathway that regulates diverse physiological functions, maintains organ homeostasis, regulates tissue repair and wound healing, and may serve as an anti-inflammatory mechanism that protects against organ inflammation and injury.

The roles of Sp1 signaling in pulmonary physiology and pathology are not well studied. Based on the functions of Sp1-regulated genes, the Sp1 pathway is expected to play important roles in maintaining organ homeostasis, in tissue repair and in anti-inflammatory response in the lungs. Sp1 activity is down-regulated in endotoxemic lungs [23], which may contribute to lung inflammation and injury.

On the other hand, the NF-κB pathway is a major inducible pathway. In contrast to Sp1 pathway, the NF-κB pathway is not activated or is minimally activated under physiological conditions, but is activated under pathological conditions. Inflammatory or stress stimuli activate I-κB kinases, which phosphorylate I-κBs, leading to I-κB degradation [24]. NF-κB dimer is then released and translocated into nucleus, where it binds to the promoter region of NF-κB target genes, leading to gene transcription. NF-κB mediates transcriptional expression of hundreds of inducible genes, most of which are pro-inflammatory and stress response genes [24]. Products of those genes are essential for immune and host defense responses, but are also key mediators of many pathological conditions, including sepsis [24], multiple organ injury [24, 25], diabetes [26], cardiovascular diseases [27] and neurological disorders [28].

Lung inflammation is initiated principally by activating pro-inflammatory transcription factors, such as NF-κB and activator protein 1 [24, 29–31]. The NF-κB signaling plays a pivotal role in endotoxemic lung inflammation and injury [24]. LPS triggers cascades of molecular and cellular events, leading to the activation of NF-κB pathway, which mediates the expression and biosynthesis of hundreds of inflammatory mediators [24]. These mediators cause the activation of leukocytes and platelets, generation of reactive oxidant species, release of proteases, and activation of coagulation pathways [24, 29–31]. These mechanisms act in concert to cause lung inflammation and injury [24, 29–31].

Crosstalk between Sp1 and NF-κB pathways can occur and may occur at multiple levels. We have previously reported that LPS down-regulated Sp1 activity in lungs by inducing or activating an LPS inducible Sp1 degrading enzyme (LISPDE) activity, which selectively degraded Sp1 protein, resulting in diminished tissue levels of Sp1 protein and activity [32]. However, the signaling pathways mediating the activation of LISPDE activity and Sp1 down-regulation remain to be elucidated. NF-κB signaling may mediate LPS-induced Sp1 down-regulation. Conversely, Sp1 signaling may counteract NF-κB-mediated inflammatory signals. Sp1 mediates the expression of multiple anti-inflammatory genes [20–22], whose products are well-known to inhibit NF-κB-mediated inflammatory response. Sp1 signaling may counter-regulate NF-κB-mediated inflammatory signals by stimulating anti-inflammatory cytokine expression.

Since the Sp1 and NF-κB pathways are two major signaling pathways, crosstalk between the two pathways can be significant and may have major impacts on overall pathological process and on the magnitude of lung inflammation. Studying crosstalk between the two pathways may gain new insights into the mechanisms of lung inflammation, and may reveal new target for developing new therapy. However, neither the crosstalk between the two signaling pathways nor the impact of the crosstalk on lung inflammation has been studied, although function and regulation of each individual pathway have been extensively studied.

In this study, we defined the role of NF-κB signaling in mediating Sp1 down-regulation, and studied the crosstalk between Sp1 and NF-κB pathways in endotoxemic lungs. We demonstrated that NF-κB signaling plays a pivotal role in LPS-induced down-regulation of Sp1 activity. Activation of NF-κB pathway suppresses Sp1-mediated anti-inflammatory mechanisms and promotes inflammation in the lungs. Conversely, Sp1 signaling inhibits NF-κB-mediated inflammatory response by stimulating anti-inflammatory gene expression. Crosstalk between NF-κB and Sp1 pathways regulates the balance between pro- and anti-inflammatory cytokines. Our data provides new insight into the regulatory mechanisms of lung inflammation.

## Materials and Methods

### Animal experiments

All animal studies were approved by Institutional Animal Care and Use Committee of the Feinstein Institute for Medical Research (Number, 2009–022), and were carried out in strict accordance with the recommendations in the Guide for the Care and Use of Laboratory Animals of the National Institutes of Health. Mice deficient in NF-B p50 gene (p50-KO) were purchased from Jackson Laboratory (Bar Harbor, ME) and were backcrossed to FVB genetic background for at least 6 generations. Wild type (WT) and p50-KO mice (25–30 g) were injected with saline (1 ml/kg, control) or *Samonelia Enteritidis* LPS (10 mg/kg, i.p.). For time course studies, lungs were harvested 5, 10, 15, 30, 60, 120 and 240 minutes after LPS injection. To study the effects of NF-B blockade, mice in WT-Con, WT-LPS, p50-KO-Con and p50-KO-LPS groups were injected with saline or LPS as described above. Lungs were harvested 2, 4 or 6 hours after LPS injection. To study the effects of the selective LISPDE inhibitor, WT mice in Con or LPS group were injected with saline or LPS as described above. Mice in TI and LPS+TI groups were injected with a peptide trypsin inhibitor (IT, 100 mg/kg, i.p.), a selective inhibitor of LISPDE activity [32], 30 minutes before saline or LPS injection. Animals were sacrificed by CO2 asphyxiation at 2, 4 or 6 hours after saline or LPS injection, and lungs harvested.

### Electrophoretic mobility shift assay (EMSA)

Nuclear protein was extracted from lungs. Sp1 and NF-B DNA binding activities were measured as we previously described [23, 33]. Sp1 (5'-ATTCGATCGGGGCGGGGCCAG-3’) and NF-κB (5’-AGTTGAGGGGACTTTCCCAGGC-3) consensus oligonucleotide probes were end-labeled with [γ-^32^P] ATP (PerkinElmer, Waltham, MA). Nuclear protein (10 μg) was incubated with 50,000 cpm ^32^P-labeled Sp1 or NF-κB probe for 30 minutes in binding buffer consisted of 10 mM Tris-Cl, pH 7.5, 1 mM MgCl_2_, 50 mM NaCl, 0.5 mM DTT, 0.5 mM EDTA, 4% glycerol and 1 μg of poly-(dI•dC). The specificity of Sp1 or NF-κB DNA binding was determined in competition reactions, in which a 50-fold molar excess unlabelled Sp1 or NF-κB, or an unrelated oligonucleotide was added to the binding reaction 10 minutes prior to the addition of radiolabeled probe. In the supershift assay, antibody against RelA/p65, RelB, c-Rel or p50, or a combination of p50+p65 (all from Santa Cruz Biotech, Dallas, TX) was added to the reaction mixture immediately after the addition of radiolabeled NF-κB probe. Reaction was subjected to non-denaturing 4% polyacrylamide gel electrophoresis. Gel was vacuum-dried and exposed to X-ray film.

### Western blot analysis

Nuclear and membrane proteins were extracted from each group of lungs. Equal amount of nuclear or membrane protein (10 or 20 μg/lane) were separated on 7.5% SDS-polyacrylamide gel under denaturing condition, and electroblotted to nitrocellulose membrane. After incubation in blocking solution (5% dry milk in TBST) at RT for 2 hours, the membrane was incubated with antibody against Sp1, eNOS or actin (Santa Cruz Biotech, Dallas, TX) at RT for 1 hour. The membrane was washed and incubated with secondary antibody conjugated to horseradish peroxidase at RT for 1 hour. Peroxidase labeling was detected using SuperSignal West Pico Kit (Thermo Fisher Scientific, Waltham, MA).

### Measurement of LPISPDE activity

Lung tissue level of LISPDE activity was measured as we previously described [32]. Nuclear protein from each group of lungs (10 g) was mixed with fuorogenic peptide, N-t-Boc-Gln-Ala-Arg-AMC (200 M), which is a specific substrate for LISPDE activity [32], in reaction buffer and incubated at RT. The rate of AMC (7-amino-4-methyl-coumarin) release was monitored for 30 minutes using spectrofluorophotometer (Shimadzu, Kyoto, Japan) at excitation of 380 nm and emission of 460 nm. Tissue level of LISPDE activity was expressed as fluorescent unit per minute per mg protein.

### Measurement of tissue levels of cytokines

Lungs were homogenized in ice-cold protein extracting buffer containing: 25 mmol/L, 0.5 mmol/L EDTA, 0.5 mmol/L EGTA, 10 mg/ml of leupeptin, 1 mmol/L pepstain and 0.1 mg/ml phenylmethylsulfonylfluoride. The homogenate was centrifuged at 12,000 *g* for 15 min, and resulting supernatant was collected as cytosolic protein. Tissue levels of TNF-α and IL-10 were measured using ELISA kits (eBioscience, San Diego, CA). The detection limit is 8 pg/mL or 32 pg/mL for TNF-α or IL-10 ELISA kit.

### Statistical Analysis

**Fig 1:**
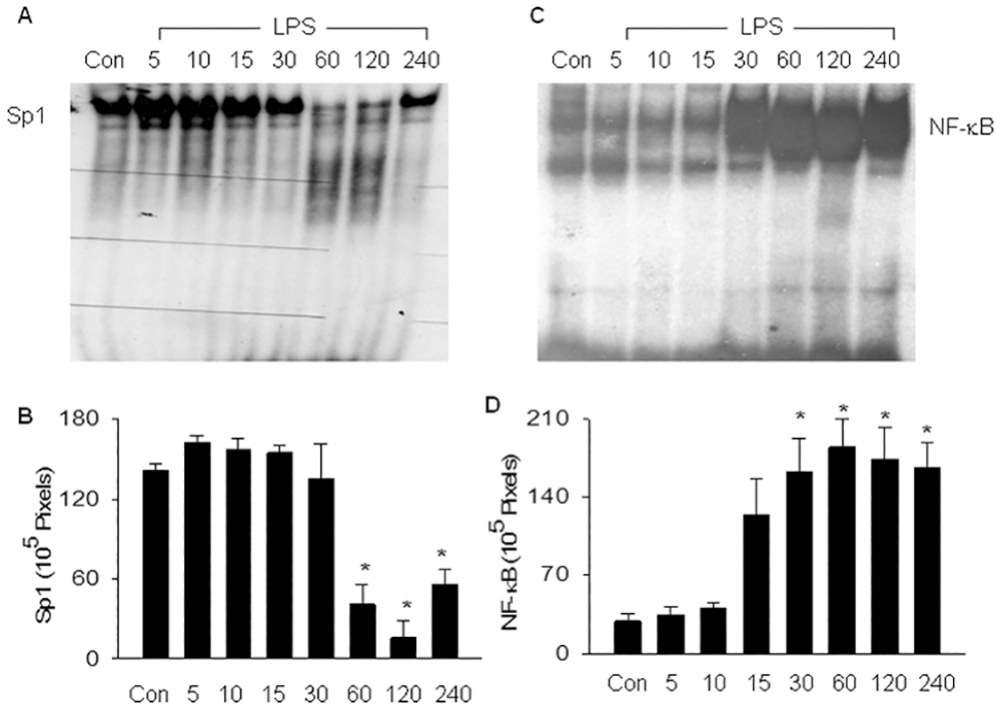
LPS-induced decrease in Sp1 DNA binding activity is associated with an increased NF-κB binding activity. Wild type (WT) mice were injected with saline (1 ml/kg, Con) or *Samonelia Enteritidis* LPS (10 mg/kg, i.p.). Lung tissue level of Sp1 (A) or NF-κB (B) DNA binding activity was measured at the indicated times (in minutes) after LPS injection, using electrophoretic mobility shift assay (EMSA). (A) and (C): EMSA photographs show that LPS-induced decrease in Sp1 DNA binding activity is associated with an increased NF-κB binding activity.(B) and (D): The EMSA Sp1 and NF-B bands were quantified using densitometry, and expressed as x10^5^ pixels. Mean ± SEM of 4 mice per group. *, p < 0.05, compared to control groups (one way ANOVA).

Data were expressed as mean ± S.E.M. Statistical analysis was performed using SigmaStat software (Systat Software, San Jose, CA) by one-way (for Fig 1) or two-way (for other Figures) ANOVA. *Post hoc* analyses were performed using Holm-Sidak method or Student-Newman-Keuls Method. Null hypothesis was rejected at 5% level.

## Results

### LPS-induced down-regulation of Sp1 activity is associated with increased NF-κB activity

To study the crosstalk between the two pathways, we first clarify if activation of Sp1 pathway and activation of NF-κB pathway are associated. We correlated the time course of LPS-induced down-regulation of Sp1 DNA binding activity to time course of LPS-induced NF-κB DNA binding activity. We have previously characterized the Sp1/DNA complex being composed mainly of Sp1 and Sp3 in this mouse lung [23]. Our previous and current competition assays confirmed the specificity of the Sp1 band (S1 Fig). We also confirmed the specificity of the NF-κB band (S1 Fig). Supershift assay showed that the NF-κB/DNA complex is composed predominantly of p50/p65 heterodimer (S1 Fig) [33].

Consistent with the housekeeping role for Sp1, tissue level of Sp1 DNA binding activity was high in control lungs. The constitutively activated Sp1 activity was slightly reduced at 30 minutes, greatly reduced or diminished at 60 and 120 minutes, and partially recovered at 240 minutes after LPS challenge (Fig 1). Tissue level of NF-κB DNA binding activity was minimal in control lungs and in lungs challenged with LPS for 5 or 10 minutes, increased in lungs challenged with LPS for 15 minutes, and significantly increased in lungs challenged with LPS for 30, 60, 120 or 240 minutes (Fig 1). The initial elevation in NF-κB DNA binding activity was observed at 15 minutes, which temporally preceded the initial decrease in Sp1 binding activity. Thus, LPS-induced down-regulation of Sp1/Sp3 binding activity was associated with an up-regulation of NF-κB (p50/p65) binding activity with latter temporally preceded the former.

### Blockade of NF-κB activation reverses LPS-induced Sp1 down-regulation

**Fig 2:**
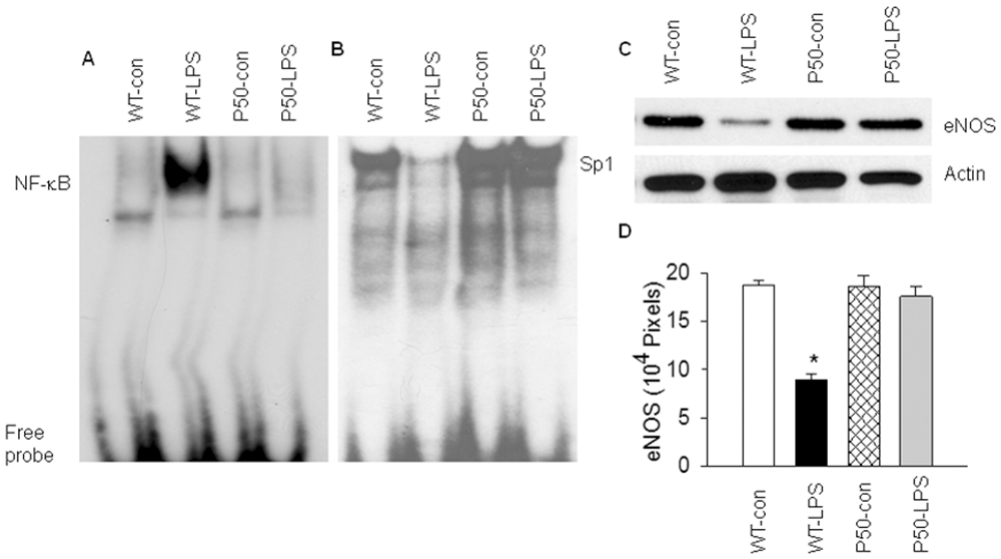
Blockade of NF-κB activation prevents LPS-induced down-regulation of Sp1 activity. WT mice or mice deficient in NF-κB p50 gene (p50-KO) were injected with saline or LPS. Lung tissue level of NF-B or Sp1 DNA binding activity was measured at 2 hours, or endothelial nitric oxide synthase (eNOS) protein was measured at 6 hours after LPS injection. (A). Representative EMSA photograph shows that p50 gene deletion abolishes LPS-induced NF-B binding activity. Representative of 3 independent experiments. (B). Representative EMSA photograph shows that blockade of NF-κB activation in p50-KO mice prevents LPS-induced down-regulation of Sp1 binding activity. Representative of 3 independent experiments. (C). Representative Western blot photographs show that NF-κB blockade in p50-KO mice mitigates LPS-induced down-regulation of eNOS expression (an indicator of Sp1 transactivation activity). (D). The Western blot eNOS bands were quantified using densitometry and expressed as x10^4^ pixels. Means ± SEM of 4 animals per group. *, p < 0.05, compared to the other 3 groups (two way ANOVA).

We next examined if blocking NF-κB activation mitigates LPS-induced Sp1 down-regulation. We blocked NF-κB activation using mice deficient in NF-κB p50 gene (p50-KO). Our supershift assay demonstrated that LPS-induced NF-κB complex is composed predominantly of p50/p65 heterodimer (S1 Fig) [33]. The lack of p50 protein in p50-KO mice diminishes or greatly reduces nuclear translocation of p50/p65 heterodimer, leading to an inhibition of NF-κB activation. Here, we confirmed that p50 gene deletion blocks NF-κB activation in this mouse model by demonstrating that LPS markedly increased NF-κB DNA binding activity in WT, but not in p50-KO mice (Fig 2). Blockade of NF-κB activity prevented LPS-induced down-regulation of Sp1 DNA binding activity in p50-KO mice (Fig 2). To verify that blockade of NF-κB activity prevents LPS-induced down-regulation of Sp1 transactivation activity, we compared lung tissue levels of eNOS protein, a putative Sp1-dependent gene product, between LPS-challenged WT and p50-KO mice. As shown in Fig 2, tissue level of eNOS protein was significantly reduced in WT (no NF-κB blockade), but not in p50-KO lungs (with NF-κB blockade) 6 hours after LPS challenge. Blockade of NF-κB activity prevented LPS-induced down-regulation of eNOS protein expression, implying that NF-κB blockade prevented LPS-induced suppression of Sp1 transactivation activity (Fig 2). These results suggest a causal relationship between LPS-induced NF-κB activation and Sp1 down-regulation.

### Blockade of NF-κB activation suppresses LISPDE activity

**Fig 3:**
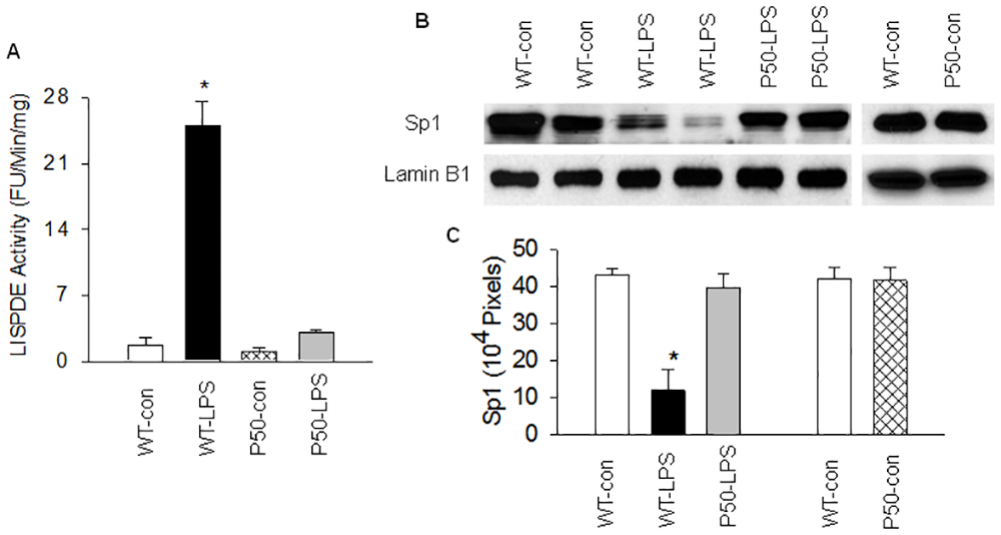
Blockade of NF-κB activation reduces LPS-inducible Sp1 degrading enzyme (LISPDE) activity and prevents Sp1 protein degradation. Nuclear level of LISPDE activity or Sp1 protein in lungs was measured in WT and p50-KO mice at 2 hours after LPS injection. (A). Bar graph shows that NF-κB blockade in p50-KO mice suppresses nuclear LISPDE activity. Means ± SEM of 5 mice per group. *, p < 0.05, compared to the other 3 groups (two way ANOVA). (B). Representative Western blot photographs show that NF-κB blockade in p50-KO mice inhibits LPS-induced nuclear Sp1 protein degradation. (C). The Western blot Sp1 bands were quantified using densitometry and expressed as x10^4^ pixels. Means ± SEM of 4 mice per group. *, p < 0.05, compared to the other groups (two way ANOVA).

We have previously demonstrated that LPS down-regulates Sp1 activity by inducing or activating an LISPDE activity, which selectively degrades Sp1 protein, resulting in a diminished tissue level of Sp1 activity [23, 32]. To study the mechanism by which NF-κB blockade mitigates LPS-induced Sp1 down-regulation, we examined the effect of NF-κB blockade on LISPDE activity. Compared to controls, nuclear proteins from LPS-challenged WT lungs had a 14.5-fold increase in LISPDE activity, which was repressed by 88% in nuclear proteins from LPS-challenged p50-KO lungs (Fig 3). Consistent with the remarkable increase in LISPDE activity, nuclear Sp1 protein content was greatly reduced in LPS-challenged WT lungs (Fig 3). By contrast, nuclear Sp1 protein content was at control level in LPS-challenged p50-KO lungs, in which nuclear LISPDE activity was greatly suppressed (Fig 3). P50 gene deletion had no effect on basal levels of LISPDE activity and Sp1 protein (Fig 3). This result suggests that NF-κB blockade in p50-KO mice prevents LPS-mediated induction or activation of LISPDE activity in the lungs.

### Inhibition of LISPDE activity restores Sp1 activity in LPS-challenged WT mice

**Fig 4:**
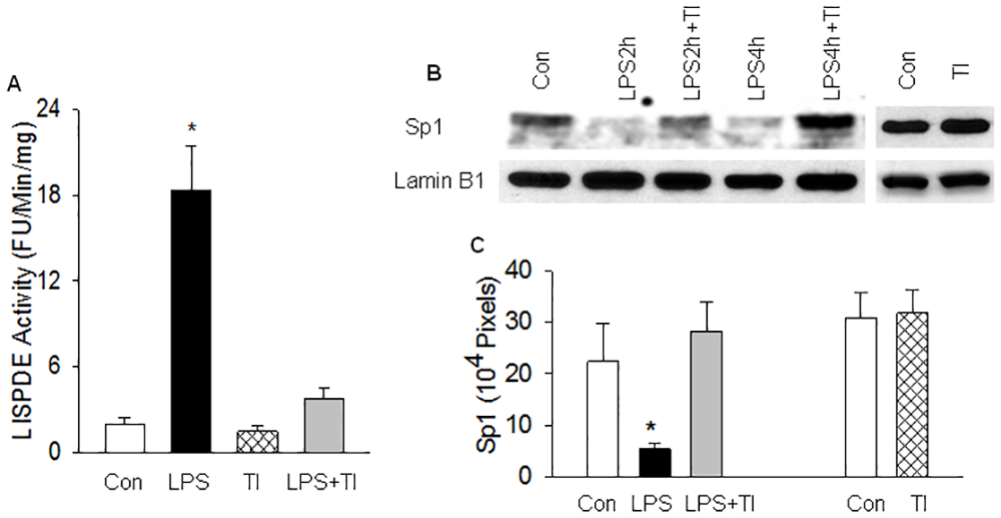
Inhibition of LISPDE activity prevents LPS-induced Sp1 protein degradation. WT mice in Con or LPS group were injected with saline or LPS, and in TI or LPS+TI group were injected with peptide trypsin inhibitor (TI, 100 mg/kg, i.p.), a selective inhibitor of LISPDE activity, 30 minutes before saline or LPS injection. Nuclear level of LISPDE activity was measured at 2 hours or Sp1 protein in lungs was measured at 2 or 4 hours after LPS injection. (A). Bar graph shows that TI remarkably inhibits nuclear LISPDE activity *in vivo*. Means ± SEM of 5 mice per group. *, p < 0.05, compared to the other groups (two way ANOVA).(B). Representative Western blot photographs show that TI prevents LPS-induced nuclear Sp1 protein degradation. LPS2h, LPS 2 hours. LPS4h, LPS 4 hours, TI, TI+saline. (C). The Western blot Sp1 protein bands were quantified using densitometry and expressed as x10^4^ pixels. Means ± SEM of 4 animals per group. *, p < 0.05, compared to the other groups (two way ANOVA).

**Fig 5:**
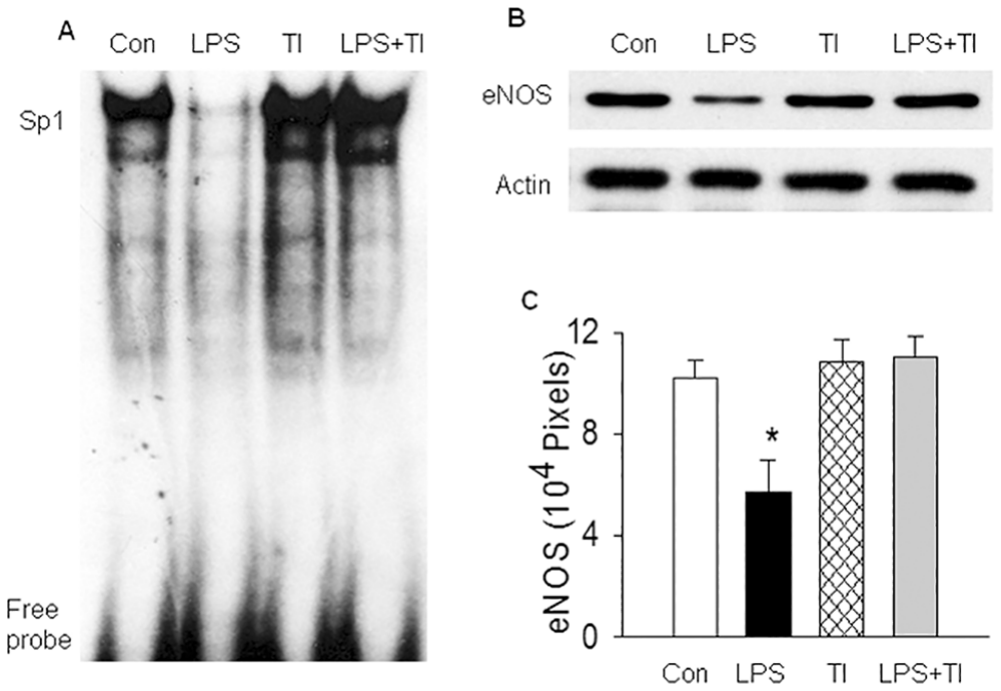
Inhibition of LISPDE activity mitigates LPS-induced down-regulation of Sp1 activity. WT mice in Con or LPS group were injected with saline or LPS, and in TI or LPS+TI group were injected with TI 30 minutes before saline or LPS injection. Lung tissue level of Sp1 DNA binding activity was measured at 2 hours or eNOS protein was measured at 6 hours after LPS injection. (A). Representative EMSA photograph shows that the selective LISPDE inhibitor, TI, mitigates LPS-induced down-regulation of Sp1 DNA binding activity. Representative of 3 independent experiments. (B). Representative Western blot photographs show that the selective LISPDE inhibitor, TI, mitigates LPS-induced down-regulation of eNOS expression (an indicator of Sp1 transactivation activity). (C). The Western blot eNOS bands were quantified using densitometry and expressed as x10^4^ pixels. Means ± SEM of 4 mice per group. *, p < 0.05, compared to the other 3 groups (two way ANOVA).

To establish a link between the inhibition of LISPDE activity and the mitigation of LPS-induced Sp1 down-regulation caused by NF-κB blockade, we examined if treatment of WT mice with a selective inhibitor of LISPDE activity mimics the effect of NF-κB blockade in preventing Sp1 down-regulation. We have previously characterized the LISPDE activity and identified a naturally occurring peptide, trypsin inhibitor (TI), as a selective inhibitor of LISPDE activity [32]. Here, we first confirmed that TI remarkably inhibited nuclear LISPDE activity in mice *in vivo* (Fig 4). We then examined the effects of TI treatment on lung tissue levels of Sp1 protein and activity. WT mice challenged with LPS showed a greatly reduced nuclear content of Sp1 protein (Fig 4), a diminished Sp1 DNA binding activity (Fig 5), and a decreased tissue level of eNOS protein (an indicator of Sp1 transactivation activity) in the lungs (Fig 5), confirming that LPS down-regulates Sp1 activity by promoting Sp1 protein degradation. Pretreatment of LPS-challenged WT mice with TI prevented LPS-induced reduction in nuclear level of Sp1 protein (Fig 4), restored Sp1 DNA binding activity (Fig 5), and restored tissue level of eNOS protein in the lungs (Fig 5). Pretreatment of control WT mice with TI had no significant effects on basal LISPDE activity and Sp1 protein level (Fig 4), and on Sp1 binding and transactivation activities (Fig 5). These results illustrate that treatment of LPS-challenged WT mice with the selective LISPDE inhibitor mimics the effect of NF-κB blockade in abrogating LPS-induced down-regulation of Sp1 activity.

### Prevention of Sp1 down-regulation rebalances the imbalance between pro- and anti-inflammatory cytokines

**Fig 6:**
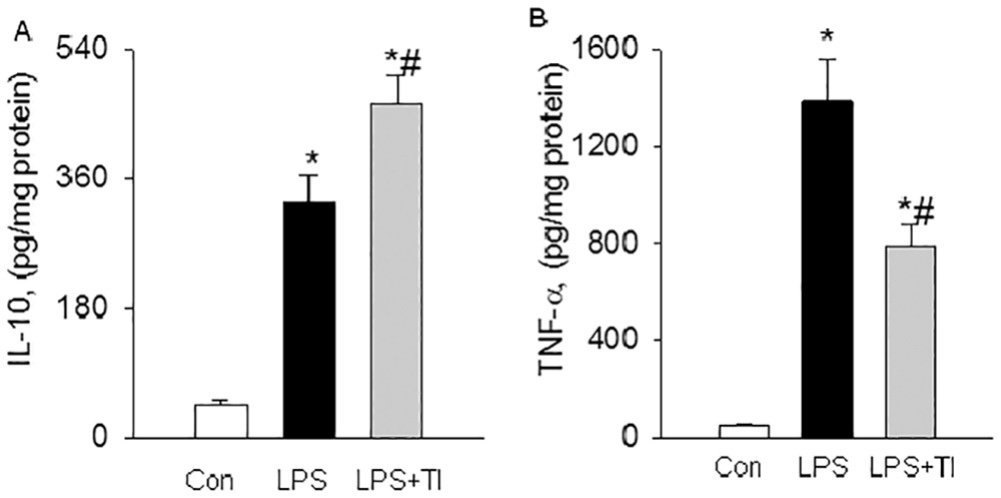
Prevention of Sp1 down-regulation rebalances the imbalance between pro- and anti-inflammatory cytokines. WT mice in Con, LPS or LPS+TI group were injected with saline, LPS, or LPS plus TI as described above. Lung tissue levels of IL-10 and TNF-α were measured at 6 hours after LPS injection. (A). Bar graph shows that prevention of Sp1 down-regulation by TI treatment augments LPS-induced lung tissue IL-10 level. Means ± SEM of 5 mice per group. *, p < 0.05, compared to Con group. #, p < 0.05, compared to LPS alone group (two way ANOVA). (B). Bar graph shows that prevention of Sp1 down-regulation by TI treatment inhibits LPS-induced tissue TNF-α level. Means ± SEM of 5 mice per group. *, p < 0.05, compared to Con group. #, p < 0.05, compared to LPS group (two way ANOVA).

To understand the pathophysiological significance of Sp1 and NF-κB crosstalk, we measured lung tissue level of TNF-α and IL-10. TNF-α is a classic pro-inflammatory cytokine and product of NF-κB-regulated gene, whereas IL-10 is a classic anti-inflammatory cytokine and product of Sp1-dependent gene. Challenge of WT mice with LPS increased lung tissue levels of TNF-α and IL-10 (Fig 6). Prevention of Sp1 down-regulation by treating LPS-challenged mice with the LISPDE inhibitor augmented tissue level of IL-10 (Fig 6), and concomitantly inhibited tissue level of TNF-α (Fig 6). This result suggests that crosstalk between NF-κB and Sp1 pathways regulates the balance between pro- and anti-inflammatory cytokines and inflammation in lungs.

## Discussion

Sp1 is a major transcription factor that mediates the expression of a large number of constitutive genes whose products play pivotal roles in many physiological processes, in housekeeping function, and in organ repair and anti-inflammatory responses. NF-κB is a key transcription factor that mediates the expression of hundreds of inducible genes whose products play critical roles in many pathophysiological processes, in disrupting organ homeostasis, and in tissue injury and inflammatory responses. Crosstalk between the two pathways could be pathophysiogically significant, but has not been studied.

This report studied the crosstalk between NF-κB and Sp1 pathways. In particular, we defined the role of NF-κB signaling in mediating LPS-induced down-regulation of Sp1 activity *in vivo*. We demonstrated that LPS-induced down-regulation of Sp1 DNA binding activity was concomitantly associated with an up-regulation of NF-κB activity with NF-κB activation preceding Sp1 down-regulation. Blockade of NF-κB activation in p50-KO mice prevented LPS-induced down-regulation of Sp1 DNA binding activity and Sp1 transactivation activity, as indicated by mitigation of LPS-induced down-regulation of eNOS protein, a putative Sp1-dependent gene product. These results suggest a causal relationship between LPS-induced NF-κB activation and Sp1 down-regulation, and define the role of NF-κB in mediating LPS-induced Sp1 down-regulation.

NF-κB is known to repress Sp1-mediated gene transcription by direct interaction between NF-κB and Sp1 proteins [34] or by protein-protein interaction via a partner protein in the transcription complex [35]. However, this direct antagonistic interaction is unlikely to be a major mechanism by which NF-κB down-regulates Sp1 activity. We have previously demonstrated that LPS had no effect on Sp1 mRNA expression [23], and demonstrated here that nuclear levels of NF-κB activity and Sp1 protein were inversely correlated in endotoxemic lungs. When LPS-induced NF-κB activity peaked (2 hours), nuclear Sp1 protein was minimally detected. Thus, there would be little Sp1 protein in the nucleus for a direct NF-κB and Sp1 protein interaction.

NF-κB is likely to act upstream of Sp1 protein. LPS down-regulates Sp1 activity by inducing or activating an LISPDE activity, which degrades Sp1 protein, resulting in a down-regulation of Sp1 activity [23, 32]. We demonstrated here that blockade of NF-κB activity in p50-KO mice remarkably inhibited LISPDE activity, prevented Sp1 protein degradation, and restored Sp1 binding activity and transactivation activities in LPS-challenged mice. Inhibition of LISPDE activity by treatment of LPS-challenged mice with a selective inhibitor of LISPDE activity mimicked the effect of NF-κB blockade in preventing Sp1 protein degradation and in restoring Sp1 binding and transactivation activities. Collectively, these results suggest that the NF-κB pathway mediates LPS-induced down-regulation of Sp1 activity by inducing or activating LISPDE activity. At present, it is technically challenging to investigate how NF-κB activation leads to the induction or activation of LISPDE activity, because the LISPDE protein has not been fully purified and its gene has not been cloned.

Depending on stimuli, disease models, and partner protein with which p50 interacts, NF-κB p50 has both pro- and anti-inflammatory actions [24, 33, 36–39], so does p50 gene deletion. P50 forms p50/p50/Bcl-3 (B-cell lymphoma 3-encoded protein) or p50/p50/histone deacetylase-1 repressor complex, which suppresses transcriptional expression of inflammatory genes [36, 37]. Under these circumstances, p50 gene deletion would exacerbate inflammation. On other hand, p50 forms p50/p65 heterodimer, which mediates the transcriptional expression of a large number of pro-inflammatory genes [24]. Under these conditions, p50 gene deletion would attenuate inflammation [24, 33, 38, 39]. We propose here that p50 gene deletion inhibits NF-κB activation by blocking the canonical NF-κB pathway, which is characterized by I-κBα degradation and nuclear translocation of p50/p65 heterodimer [24]. We have previously demonstrated that I-κBα degradation is necessary for LPS-induced NF-κB activation [24, 33]. We and others have demonstrated that I-κBα overexpression blocked LPS-induced NF-κB activation [40, 41]. Our supershift assay revealed that LPS-induced DNA/NF-κB complex is composed predominantly of p50/p65 heterodimer, and contains minimal p50/p50 homodimers. We demonstrated that p50 gene deletion diminished LPS-induced NF-κB band. It is likely that the lack of p50 protein in p50-KO mice diminishes nuclear translocation of p50/p65 heterodimer, leading to an inhibition of NF-κB activation. NF-κB blockade in turn inhibits the induction or activation of LISPDE activity, preventing Sp1 protein degradation and subsequent down-regulation of Sp1 activity.

Sp1 signaling appears to inhibit NF-κB-mediated inflammatory signals. We observed that enhancing tissue level of Sp1 activity by pretreatment of LPS-challenged mice with the LISPDE inhibitor, TI, repressed tissue level of TNF-α in the lungs. Since TNF-α is a major effector molecule in NF-κB signaling pathway, this result suggests that Sp1 signaling inhibits NF-κB inflammatory signaling.

TNF-α is a product of NF-κB-dependent gene. However, TI is unlikely to suppress TNF-α secretion by directly inhibiting NF-κB-mediated TNF-α gene expression. LPS induces TNF-α gene transcription by activating the canonical NF-κB pathway, whose activation is controlled by I-κBα degradation [24, 40, 41]. However, neither LISPDE activity nor TI has an effect on I-κBα degradation [23, 32], implying that TI is unlikely to directly inhibit LPS-induced NF-κB activity.

One likely explanation is that TI stimulates Sp1 activity, which represses lung tissue TNF-α production. TI inhibits LISPDE activity and the subsequent degradation of Sp1 protein, resulting in an increased tissue level of Sp1 activity. An increased Sp1 activity can inhibit NF-κB-mediated inflammatory response, including TNF-α secretion, by several mechanisms. First, Sp1 mediates the transcription of a large number of constitutive genes, many of whose products have anti-inflammatory action. For example, Sp1 mediates eNOS expression [9]. It is well documented that eNOS-generated NO protects against inflammation induced by various pathological insults [42–44]. NO inhibits NF-κB activity [45, 46], suppresses pro-inflammatory cytokine [45, 47] and adhesion molecule expression [45], and reduces neutrophil migration into organs [48], limiting organ inflammation and injury [49, 50].

Second, Sp1 mediates or contributes to the transcriptional expression of multiple anti-inflammatory genes, including IL-10 [20], suppressor of cytokine signaling 3 [21], peroxisome proliferator-activated receptor-α [22] and leukemia inhibitory factor [51]. These anti-inflammatory mediators are important negative regulator of NF-κB inflammatory pathway and other pro-inflammatory pathways [52–54], and act to counter-regulate inflammatory response [52–54]. For example, IL-10 suppresses I-κB kinase activity, blocks NF-κB activation [55, 56], reduces pro-inflammatory mediator production [55, 56], improves bacteria-induced lung inflammation [57], and protects animals against lethal endotoxemia [58] and sepsis [59]. Collectively, TI pretreatment of LPS-challenged mice prevents Sp1 protein degradation and enhances tissue level of Sp1 activity, resulting in an increased expression of anti-inflammatory cytokines, which inhibits LPS-induced TNF-α expression. In supporting this explanation, we demonstrated here that TI treatment increased tissue level of IL-10 and concomitantly decreased tissue level of TNF-α. Others have demonstrated that IL-10 represses TNF-α expression [60, 61].

We demonstrated that NF-κB mediates LPS-induced down-regulation of Sp1 activity. Since Sp1 activity is an endogenous anti-inflammatory mechanism, this result suggests that NF-κB activation can promotes lung inflammation by suppressing Sp1-mediated anti-inflammatory mechanisms. We have revealed a new mechanism by which NF-κB activation promotes lung inflammation and injury.

In summary, we demonstrated a crosstalk between NF-κB and Sp1 pathways *in vivo*. First, NF-κB mediates LPS-induced Sp1 down-regulation. LPS activates NF-κB, which induces or activates the LISPDE activity that degrades Sp1 protein, resulting in a diminished Sp1 activity. Blockade of NF-B activation in p50-KO mice inhibited LISPDE activity, prevented Sp1 protein degradation, and restored tissue levels of Sp1 binding and transactivation activities. Second, Sp1 signaling is a negative regulator of NF-κB-mediated inflammatory pathways. Enhancing tissue level of Sp1 activity by pretreatment of LPS-challenged mice with TI increased tissue level of IL-10 and decreased tissue level of TNF-α in the lungs. Crosstalk between NF-B and Sp1 pathways regulates the balance between pro- and anti-inflammatory cytokines and the magnitude of lung inflammation.

## Supporting Information

S1 Fig Representative electrophoretic mobility shift assay autoradiographs show the characteristics of LPS-induced NF-κB DNA complex and the specificity of Sp1 DNA binding in mouse lungs. (PDF)
