# Supplementary material for: LPS Down-Regulates Specificity Protein 1 Activity by Activating NF-κB Pathway in Endotoxemic Mice
Source: PLoS One. 2015 Jun 23;10(6):e0130317. doi: 10.1371/journal.pone.0130317 (PMC4478004; doi:10.1371/journal.pone.0130317)
Supplement: S1 Fig — (PDF) [file pone.0130317.s001.pdf]

## Supporting Information

**S1 Figure S1**

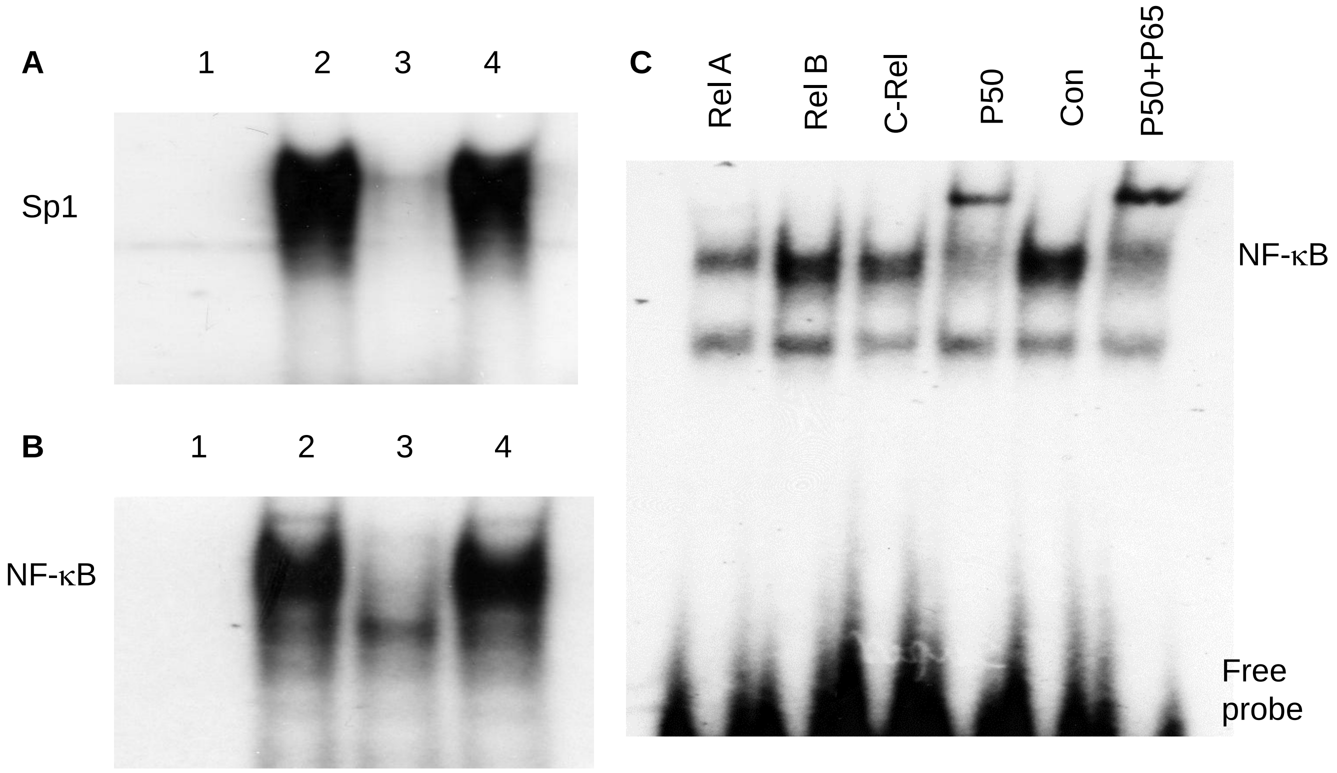

**Figure S1.** Representative electrophoretic mobility shift assay autoradiographs show the characteristics of LPS-induced NF-κB-DNA complex and the specificity of Sp1 DNA binding in mouse lungs.

**A:** Competition assay shows the specificity of Sp1 DNA binding. Lane 1, Sp1 probe without nuclear extract; lane 2, nuclear extract from control lungs; lanes 3 and 4, the same sample as in lane 2 but including a 50-fold molar excess of unlabeled Sp1 probe (lane 3) or NF-κB probe (lane 4).

**B:** Competition assay shows the specificity of NF-κB DNA binding. Lane 1, NF-κB probe without nuclear extract; lane 2, nuclear extract from LPS-challenged lungs; lanes 3 and 4, the same sample as in lane 2 but including a 50-fold molar excess of unlabeled NF-κB probe (lane 3) or activating protein-2 probe (lane 4).

**C:** Supershift assay using nuclear extract from LPS-challenged lungs. NF-κB DNA binding reaction was carried out in the absence (Con) and presence of antibodies to RelA/p65, RelB, c-Rel, p50 or a combination of p50+p65. The NF-κB band was reduced (shifted) by p50 or p65 antibody, slightly reduced by c-Rel antibody, and was diminished by p50+p65 antibodies, indicating that the NF-κB DNA binding complex is composed predominantly of NF-κB p50 and p65 subunits.
